# Influenza A Virus Infection Damages Zebrafish Skeletal Muscle and Exacerbates Disease in Zebrafish Modeling Duchenne Muscular Dystrophy

**DOI:** 10.1371/currents.md.8a7e35c50fa2b48156799d3c39788175

**Published:** 2017-10-25

**Authors:** Michelle Goody, Denise Jurczyszak, Carol Kim, Clarissa Henry

**Affiliations:** Department of Molecular and Biomedical Sciences, University of Maine, Orono, ME 04469, USA; Graduate School for Biomedical Sciences and Engineering, School of Biology and Ecology, University of Maine. Orono, Main, USA

## Abstract

**INTRODUCTION:**

Both genetic and infectious diseases can result in skeletal muscle degeneration, inflammation, pain, and/or weakness. Duchenne muscular dystrophy (DMD) is the most common congenital muscle disease. DMD causes progressive muscle wasting due to mutations in Dystrophin. Influenza A and B viruses are frequently associated with muscle complications, especially in children. Infections activate an immune response and immunosuppressant drugs reduce DMD symptoms. These data suggest that the immune system may contribute to muscle pathology. However, roles of the immune response in DMD and Influenza muscle complications are not well understood. Zebrafish with dmd mutations are a well-characterized model in which to study the molecular and cellular mechanisms of DMD pathology. We recently showed that zebrafish can be infected by human Influenza A virus (IAV). Thus, the zebrafish is a powerful system with which to ask questions about the etiology and mechanisms of muscle damage due to genetic and/or infectious diseases.

**METHODS:**

We infected zebrafish with IAV and assayed muscle tissue structure, sarcolemma integrity, cell-extracellular matrix (ECM) attachment, and molecular and cellular markers of inflammation in response to IAV infection alone or in the context of DMD.

**RESULTS:**

We find that IAV-infected zebrafish display mild muscle degeneration with sarcolemma damage and compromised ECM adhesion. An innate immune response is elicited in muscle in IAV-infected zebrafish: NFkB signaling is activated, pro-inflammatory cytokine expression is upregulated, and neutrophils localize to sites of muscle damage. IAV-infected dmd mutants display more severe muscle damage than would be expected from an additive effect of dmd mutation and IAV infection, suggesting that muscle damage caused by Dystrophin-deficiency and IAV infection is synergistic.

**DISCUSSION:**

These data demonstrate the importance of preventing IAV infections in individuals with genetic muscle diseases. Elucidating the mechanisms of immune-mediated muscle damage will not only apply to DMD and IAV, but also to other conditions where the immune system, inflammation, and muscle tissue are known to be affected, such as autoimmune diseases, cancer, and aging.

## INTRODUCTION

Skeletal muscle is critical for homeostasis because skeletal muscle is required for breathing, posture, locomotion, metabolism, thermoregulation, and the immune response. Muscle tissue is remarkably plastic and can increase or decrease in mass in response to genetic and environmental factors. Muscle degeneration is a serious health issue that can reduce lifespan and quality of life. Muscle wasting can be caused by aging, injury, disuse, medications, genetic mutations, and infectious or inflammatory diseases. Understanding how muscle growth, regeneration, and degeneration are regulated in response to genetic and environmental insults alone and in combination is an important undertaking in order to be able to promote muscle health in cases of sickness and disease.

Skeletal muscle damage occurs in response to some genetic and infectious or inflammatory diseases. The most common, genetic muscle wasting disease is Duchenne Muscular Dystrophy (DMD), which is caused by mutations in the *DMD* gene. The most common viral infections that cause muscle pain and weakness are Influenza A and B viruses^1^ . Many strides have been made towards elucidating the mechanisms of muscle degeneration due to *DMD* mutations. Dystrophin is required in muscle fibers for sarcolemma integrity^2^ and in muscle stem cells for proper polarity and asymmetric cell division^3^. However, much less is known about the etiology of Influenza-induced muscle damage and nothing is known about the consequences of Influenza infection in the context of patients with genetic muscle wasting diseases.

Biopsies from patients with genetic muscle diseases or muscle complications of Influenza infection show biomarker and histological similarities, suggesting that these conditions may share common mechanisms of muscle damage. Creatine kinase was upregulated and correlated with poor outcome in patients with IAV muscle complications^4^. Creatine kinase upregulation is also used in the diagnosis of DMD. The first histological report of muscle biopsies from IAV (H1N1)-infected people found muscle necrosis, fibers with variable diameters, atrophic round fibers, atrophic angulated fibers, type 1 and 2 fiber atrophy, and type 1 fiber predominance^5^. The findings from these biopsies are similar to reports of DMD histopathology, which include fiber size variability, fiber necrosis, regeneration, inflammation, and connective tissue deposition^6^. It is not known whether Influenza infection exacerbates muscle damage in the context of genetic muscle diseases.

Here, we characterize muscle damage and assay innate immune/inflammatory markers in muscle in response to IAV infection *in vivo*. We also determine the consequences of IAV infection on muscle degeneration in an animal model of DMD. To our knowledge, IAV has never been experimentally induced in any model of DMD. We used zebrafish embryos for these experiments as it has been shown that zebrafish embryos can be infected with human isolates of IAV^7^ and *sapje/dmd* mutant zebrafish are a well-characterized model that recapitulates aspects of human disease and has greatly contributed to our knowledge of DMD mechanisms and potential treatments^8^^,^^9^^,^^10^^,^^11^^,^^12^^,^^13^^,^^14^^,^^15^^,^^16^^,^^17^. Therefore, zebrafish embryos are well-suited for the study of the combinatorial effects of genetic and infectious diseases on muscle degeneration. We find that systemic IAV infection potentiates fiber damage accompanied by markers of heightened inflammation in zebrafish muscle tissue. We also find that IAV infection greatly exacerbates the extent of fiber damage in zebrafish modeling DMD. Taken together, these results show an important gene-environment interaction between the pro-inflammatory innate immune response and the *DMD* gene in skeletal muscle.

## MATERIALS AND METHODS

**Ethics statement**

Zebrafish (*Danio rerio*) used in this study were handled in accordance with the recommendations in the Guide for the Care and Use of Laboratory Animals of the National Institutes of Health. The protocols used in this study were approved by the Institutional Animal Care and Use Committee (IACUC) at the University of Maine (Protocol Number: A2013-06-03).

**Zebrafish care, staging, and husbandry**

Zebrafish were maintained in the Zebrafish Facility at the University of Maine, Orono. The facility was maintained according to IACUC standards. IACUC approved guidelines for zebrafish care followed the standard procedures (www.zfin.org) of a 14 hour light, 10 hour dark cycle at 28°C. Embryos were obtained from natural spawnings of these adult fish and grown at 28°C or 33°C. Fertilized eggs were collected and raised in egg water (60 μg/ml Instant Ocean sea salts; Aquarium Systems, Mentor, OH). Developmental staging was performed according to^18^. Transgenic lines used were the *Tg(fli1:GFP) *line^19^, *Tg(NFkB:EGFP)^nc1^* line^20^, and the *Tg(BACmpo:gfp)^i114^* line^21^. The mutant line used was the *sapje^ta222a^* line^8^. Approximately 900 embryos were used for these studies.

**IAV and Evans Blue Dye (EBD) injection**

Influenza A/PR/8/34 (H1N1) virus was purchased from Charles River Laboratories, aliquoted upon arrival, and stored at -80°C. Embryos were manually dechorionated at 2 days post fertilization (dpf) with fine forceps (DuMont no. 5). Prior to injections, 2 dpf fish were anesthetized in tricaine solution and lined up on a 3% agarose gel in a Petri dish before being injected into the Duct of Cuvier (DC) with 1.5 nl (~1×10^4^ EID_50_) of wild-type A/PR/8/34 IAV or 4 nl [~6×10^2^ plaque forming units (PFU) per embryo] of NS1-GFP A/PR/8/34 IAV in PBS with a final concentration of 0.25% phenol red. For experiments involving EBD, phenol red volume was replaced with EBD^2^^,^^22^ and zebrafish were injected as before. Sterile PBS including phenol red or EBD was injected into the DC of 2 dpf control zebrafish. Injection volumes were calibrated using a micrometer slide. Following injection, zebrafish were maintained at 33°C until fixation or euthanasia and egg water was changed daily. Euthanasia was carried out by immersion in 300mg/L MS222 buffered with Sodium Bicarbonate for 10 minutes. Microinjection was controlled by an MPPI-2 pressure microinjector (Applied Scientific Instruments) and pulled microcapillary pipettes (Sutter Instruments, Novato, CA) were used to inject the virus or PBS.

**Phalloidin staining and immunohistochemistry**

Alexa Fluor 488 phalloidin (Molecular Probes) staining involved fixing embryos in 4% Paraformaldehyde (PFA) for 4 hours (h) at room temperature (RT), washing 5 times for 5 min each (5 × 5) in PBS-0.1% Tween20, permeabilizing for 1.5h in PBS-2% Triton, washing 5×5 and then incubating in phalloidin (1:20) for 1–4h at RT or overnight at 4°C. All antibodies (Abs) were diluted in block (5% w/v Bovine Serum Albumin (BSA) in Phosphate Buffered Saline (PBS) with 0.1% Tween20). Ab staining followed phalloidin staining or started with blocking for 1h at RT, incubating in 1° Ab overnight at 4°C, washing for 2–8h in block at RT, incubating in 2° Ab overnight at 4°C, then washing for at least 1h in PBS-0.1% Tween. 1° Abs: anti-β-Dystroglycan 1:50 (Novocastra); anti-Dystrophin 1:50 (Sigma D8043); anti-Paxillin 1:50 (BD Biosciences). 2° Abs: GAM/GAR 488, 546, 633 1:200 (Invitrogen).

**Imaging**

Images were obtained on a Zeiss Imager-Z compound microscope with ApoTome attachment running Axiovision software or an Olympus Fluoview IX-81 inverted microscope with FV1000 confocal system. Linear adjustments were made to images in Adobe Photoshop and figures were collated in Adobe Illustrator. Fixed and stained zebrafish were deyolked and mounted in PBS for imaging. Live imaging for EBD experiments involved anesthetizing zebrafish in tricaine embedding them in low melt agarose and imaging in 24 well plates with glass bottoms.

**Quantitative polymerase chain reaction (qPCR)**

Total RNA was extracted from whole embryos at 24 hours post infection (hpi) by homogenizing 10 fish, treating with TRIzol reagent (Invitrogen, Carlsbad, CA) and subsequently storing at −80°C. RNA was extracted according to the manufacturer’s protocol. Reverse transcription reactions to synthesize cDNA were performed according to manufacturer’s instructions using Bio-Rad iScript reagents (Bio-Rad Laboratories, Hercules, CA). Bio-Rad SsoFast EvaGreen reagents were used for qPCR reactions according to manufacturer’s instructions. qPCR was performed on a Bio-Rad I-cycler IQDetection system using cycling parameters described previously^23^. Gene expression was normalized to the corresponding *beta-actin* value using the delta delta ct method to determine relative transcript abundance.

**Survival**

Zebrafish embryos were injected and maintained as described above. For mortality experiments, egg water was changed daily and mortality (defined as lack of a discernible heart beat) was monitored and recorded from 0-5 days post infection (dpi). Deceased zebrafish were removed each day. The *sapje* phenotype of disrupted birefringence was determined in live zebrafish using two polarized light filters.

## RESULTS

**IAV infects muscle cells and causes muscle damage**

Symptoms of IAV muscle complications include pain, tenderness, weakness, and problems with ambulation. These symptoms usually resolve in a week regardless of treatment; however, muscle complications can be severe. IAV has been shown to directly infect cultured human muscle cells24. Despite this observation, IAV is only rarely recovered from muscle biopsies^25^ and muscle biopsies show inconsistent infiltration of immune cells. Therefore, it is unclear to what degree muscle fiber infection, inflammation, or both contribute to the muscle complications caused by IAV infection. To study the effects of IAV infection on skeletal muscle tissue *in vivo*, we utilized the zebrafish model. We previously showed that IAV infects and replicates in zebrafish embryos when administered via intravenous injection^7^. We used this model of IAV infection to ask whether IAV can enter and infect muscle cells and the effect of IAV infection on muscle tissue.

To investigate the effect of IAV infection on skeletal muscle structure, 2 dpf zebrafish were infected with human IAV (H1N1, A/PR/8/34), fixed, and then stained with phalloidin to visualize F-actin at 24 and 48 hpi (hours post infection). Phalloidin staining in PBS-injected zebrafish at 24 and 48 hpi revealed the normal segmented, highly ordered, parallel arrays of muscle fibers (Fig. 1A-B). Zebrafish infected with IAV displayed foci of muscle degeneration at 24 hpi and 48 hpi (hours post infection) (Fig. 1C-D, white arrowheads). Muscle damage worsened over time: damage was more frequently observed in infected zebrafish at 48 hpi than 24 hpi (Fig. 1C-E). Sites of muscle damage were more prevalent in the anterior muscle segments than the posterior muscle segments of infected zebrafish at 24 hpi (Fig. 1F). These data show that injection of IAV into the bloodstream of zebrafish embryos has an impact on skeletal muscle tissue and results in areas of muscle degeneration; however, the data do not determine if the muscle damage observed is an indirect consequence of a systemic Influenza infection or if muscle cells are infected by IAV.

To determine if IAV infects zebrafish muscle cells, we injected 2 dpf zebrafish with a fluorescent reporter strain of IAV (NS1:GFP) where GFP expression signifies that translation of a recombinant viral gene occurred in an infected host cell^7^^,^^26^. Injection of this strain of IAV into zebrafish embryos resulted in punctate GFP fluorescence throughout embryos by 24 hpi whereas GFP fluorescence was not observed in PBS-injected control zebrafish embryos (Fig. 1G-H). To determine if any of the cell types expressing NS1:GFP were muscle cells, we imaged infected zebrafish under higher magnification. We occasionally observed zebrafish muscle cells expressing NS1:GFP (Fig. 1I-I1). The NS1:GFP fluorescent reporter strain of IAV is approximately ten-fold less infectious than wild-type IAV^26^. Therefore, infection of zebrafish with NS1:GFP did not cause foci of muscle damage and we were unfortunately unable to determine whether fiber degeneration was limited to IAV-infected muscle fibers or not. Our results show that IAV can enter and infect zebrafish muscle cells *in vivo* and suggest that muscle degeneration, pain, and weakness may be, at least in part, due to direct infection of muscle cells by IAV.

**Human IAV infects zebrafish muscle cells and causes muscle fiber damage:**
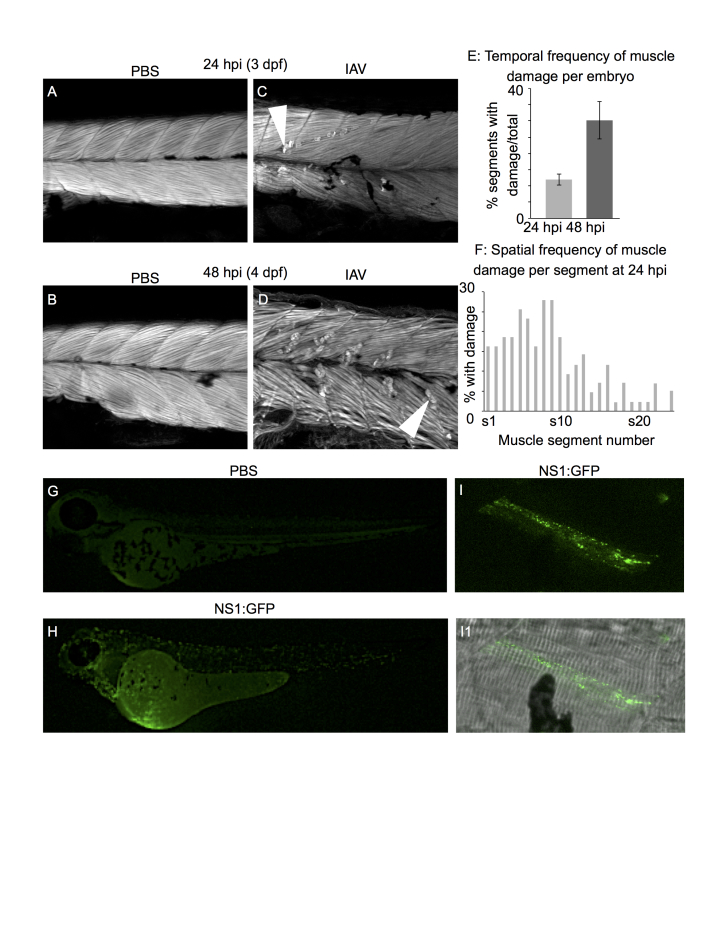
All embryo images are side mounts, dorsal top, anterior left. Panels A-D are phalloidin stained to visualize F-actin. White arrowheads denote retracted fibers. (A) PBS-injected control at 24 hpi (3 dpf). (B) PBS-injected control at 48 hpi (4 dpf). (C) IAV-infected embryo at 24 hpi. (D) IAV-infected embryo at 48 hpi. (E) Quantification of the proportion of muscle segments per embryo with damaged fibers in IAV-infected embryos over developmental time. Fiber damage is more frequently observed at 48 hpi than at 24 hpi. (F) Spatial location of damaged fibers along the anterior-posterior axis of IAV-infected embryos at 24 hpi. The frequency of damaged fibers peaks in segments 5-9, which is in the anterior of the fish near the Duct of Cuvier (the site of injection). (G) PBS-injected control at 24 hpi (3 dpf). (H) NS1-GFP-injected zebrafish at 24 hpi. Note the punctate green fluorescence in infected cells throughout the body. (I) NS1-GFP-injected zebrafish at 24 hpi. Higher magnification view of a GFP-positive, infected muscle fiber. (I1) Merged panel of NS1-GFP and brightfield images.

**IAV infection results in fiber detachment and decreased sarcolemma integrity**

We showed that IAV infection causes muscle degeneration in zebrafish. The phenotype in IAV-infected zebrafish is similar to what is seen in zebrafish models of congenital muscle diseases. Work in these zebrafish disease models showed that detached muscle fibers can occur via at least two different etiologies: (1) loss of muscle membrane (sarcolemma) integrity and subsequent fiber death, or (2) detachment of muscle fibers from their surrounding ECM prior to loss of sarcolemma integrity and cell death. Injection of the fluorescent, cell impermeable Evans blue dye (EBD) can be used to discriminate between these two possibilities. If EBD penetrates long and/or retracted muscle fibers, sarcolemma damage has occurred. Loss of sarcolemma integrity occurs in zebrafish models of DMD and dysferlinopathy^2^^,^^27^; however, retracted fibers do not take up EBD in a zebrafish model of Merosin-deficient congenital muscular dystrophy (MDC1A)^28^. Interestingly, in the two zebrafish models of primary dystroglycanopathy, each caused by a different mutation in the *dag1* gene, retracted fibers were reported to remain impermeable to EBD^29^ or to take up EBD only after retraction^30^. We sought to determine the etiology of muscle fiber retraction in IAV-infected zebrafish using the EBD permeability assay.

For this experiment, 2 dpf zebrafish were infected with IAV as before except that the phenol red in the injection solution was replaced with EBD. Live zebrafish were imaged at 24 hpi. In PBS-injected *Tg(fli1:GFP)* zebrafish, where GFP expression driven by an endothelial-specific promoter allows for visualization of the vasculature *in vivo*^19^, EBD remained within blood vessels (Fig. 2A, white arrowheads from top to bottom point to EBD in intersomitic vessels, the dorsal aorta, and the caudal vein). Uptake of EBD into muscle cells was not observed in PBS-injected zebrafish (Fig. 2A-C). In IAV-infected *Tg(fli1:GFP)* zebrafish, EBD leaked out of the vasculature and was taken up by long muscle fibers (Fig. 2D, F, black arrowheads). In addition to permeating some long muscle fibers, EBD was taken up by retracted fibers in IAV-infected zebrafish (Fig. 2E-F, white arrowheads denote EBD in retracted fibers). These data show that IAV infection increases vascular permeability as well as compromises sarcolemma integrity in attached and retracted fibers in zebrafish embryos.

**IAV infection compromises the sarcolemma:**
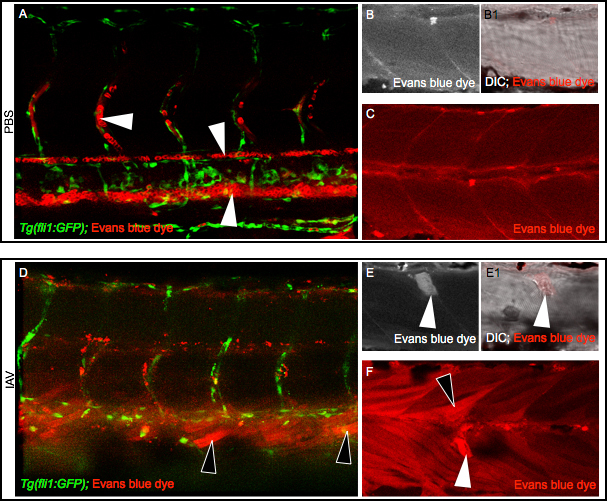
All embryo images are side mounts, dorsal top, anterior left. (A) Tg(fli1:GFP) zebrafish embryo with labeled endothelial cells (green) DC-injected with PBS plus EBD (red). EBD remains in the vasculature. White arrowheads point to EBD in an intersomitic vessel (top left), the dorsal aorta (middle), and the caudal vein (bottom right). (B-C) Wild-type zebrafish injected with PBS plus EBD. (B) Cropped EBD panel. (B1) Cropped EBD and brightfield panels merged. (C) EBD panel. Note that muscle fibers are impermeable to EBD in PBS-injected zebrafish. (D) Tg(fli1:GFP) zebrafish embryo DC-injected with IAV plus EBD (red). EBD leaked out of the vasculature and penetrated muscle fibers (black arrowheads). (E-F) Wild-type zebrafish injected with IAV plus EBD. (E) Cropped EBD panel. (E1) Cropped EBD and brightfield panels merged. (F) EBD panel. Note the uptake of EBD by long (black arrowheads) and retracted fibers (white arrowheads) indicative of sarcolemma damage.

To investigate the possibility that the detached fibers in IAV-infected zebrafish could also be due to disrupted adhesion to the ECM, we performed antibody staining to visualize intracellular proteins that localize to the myotendinous junction (MTJ) and are known to be involved in stable muscle fiber-ECM attachments. We assessed the localization of beta-Dystroglycan, Dystrophin, and Paxillin proteins in the muscle fibers of IAV-infected zebrafish. In mock-infected zebrafish, no retracted fibers were observed (Fig. 3A) and beta-Dystroglycan localized to MTJs (white arrow in Fig. 3A1-2) and to neuromuscular junctions (white arrowhead in Fig. 3A1-2). In IAV-infected zebrafish, detached fibers were clearly visible with phalloidin staining (white arrows in Fig. 3B, C, D). Many retracted fibers in IAV-infected zebrafish were observed to have beta-Dystroglycan, Dystrophin, or Paxillin still localized to their detached end (white arrowheads in Fig. 3B1-2, C1-2, D1-2). These data show that some fibers can retract in IAV-infected zebrafish without disruption to the localization of their intracellular MTJ anchoring complexes. These data are consistent with previous experiments that show fiber detachment and then death can be due to an extracellular disruption in adhesion. Altogether, our results suggest that IAV infection damages and causes muscle fiber death in zebrafish via at least two (not necessarily mutually exclusive) mechanisms: (1) loss of sarcolemma integrity and (2) failure of muscle fiber-ECM adhesion external to the sarcolemma.

**IAV infection disrupts muscle fiber-ECM adhesion:**
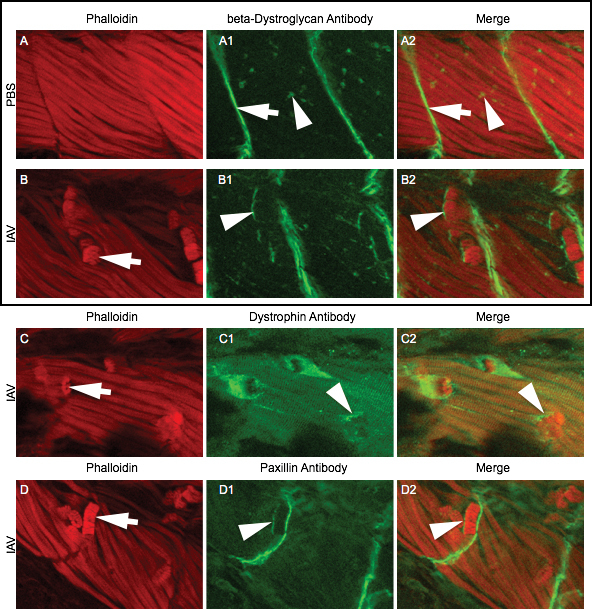
All embryo images are side mounts, dorsal top, anterior left of zebrafish at 24 hpi. Lettered panels show phalloidin staining for F-actin in red. Panels numbered 1 show immunohistochemistry for beta-Dystroglycan, Dystrophin, or Paxillin proteins in green. Panels numbered 2 are merged images of phalloidin and antibody staining. (A-A2) Phalloidin and beta-Dystroglycan staining in a PBS-injected zebrafish. White arrow in A1 denotes MTJ localized beta-Dystroglycan and white arrowhead in A1 points to neuromuscular junction localized beta-Dystroglycan. (B-B2) Phalloidin and beta-Dystroglycan staining in an IAV-injected zebrafish. White arrow in B points to a retracted fiber and white arrowheads in B1-2 highlight beta-Dystroglycan staining at the unattached end of a retracted fiber. (C-C2) Phalloidin and Dystrophin staining in an IAV-injected zebrafish. White arrow in C points to a retracted fiber and white arrowheads in C1-2 highlight Dystrophin staining at the unattached end of a retracted fiber. (D-D2) Phalloidin and Paxillin staining in an IAV-injected zebrafish. White arrow in D points to a retracted fiber and white arrowheads in D1-2 highlight Paxillin staining at the unattached end of a retracted fiber. These results showing that some retracted fibers retain the localization of ECM adhesion proteins suggest that muscle fibers-ECM adhesion can be disrupted external to the sarcolemma.

**An inflammatory innate immune response is elicited in muscle tissue upon IAV infection**

Although we have demonstrated that human IAV infects zebrafish muscle cells and causes muscle fiber damage and degeneration, it is still unclear to what degree the innate immune/inflammatory response contributes to muscle complications of IAV infection. This is because of inconsistent detection of immune cells in muscle biopsies from IAV-infected humans. The distinction between IAV-induced myopathy (muscle degeneration due to a defect within muscle) and IAV-induced myositis (muscle degeneration due to the pro-inflammatory innate immune response) is an important one to make because these conditions would likely respond to different treatments. The immune response is well conserved between humans and zebrafish (in terms of molecules, signaling pathways, and cell types/functions); however, only the innate immune response is functional in zebrafish at the time points of our experiments^31^. We have previously shown that systemic IAV infection elicits an innate immune response in zebrafish embryos/larvae^7^^,^^32^, but inflammation in muscle tissue was not specifically examined.

We used the accessibility of the zebrafish model to ask whether IAV elicits an innate immune response in zebrafish muscle cells by infecting in multiple transgenic lines of zebrafish. First, we used the NFkB reporter line^20^ to investigate activation of NFkB-dependent, pro-inflammatory innate immune signaling cascades at the molecular level. Transgenic embryos (2 dpf) were injected with PBS or IAV as before and then imaged for GFP at 24 hpi. In PBS-injected embryos, GFP expression was observed in certain cell types, such as neuromasts of the lateral line, but not in muscle cells (Fig. 4A). This result shows that NFkB signaling is normally not active in zebrafish muscle cells at 3 dpf. In IAV-injected zebrafish, in addition to the basal level of active NFkB signaling seen in controls, we observed GFP-positive muscle fibers (white arrowhead in Fig. 4B). Muscle fibers expressing GFP occurred throughout the anterior-posterior axis of infected zebrafish; however, GFP-positive muscle fibers were more prevalent in the anterior muscle segments (Fig. 4B). This distribution of GFP-positive muscle fibers correlates with the increased frequency of muscle degeneration in anterior muscle segments of IAV-infected zebrafish (Fig. 1F). Our data show that NFkB transcription factor-dependent, pro-inflammatory innate immune signaling cascades are turned on in muscle cells in IAV-infected zebrafish.

We additionally interrogated the innate immune response at the molecular level by performing qPCR to assess pro-inflammatory cytokine gene expression. We previously showed that IAV infection elicits an antiviral innate immune response in zebrafish (as assayed by differential expression of Interferon and Myxovirus Resistance Gene A family members)^7^. Here, we found that IAV-infected zebrafish had approximately 11-fold more *interleukin 1, beta* mRNA and 3.5-fold more *interleukin 8* mRNA than mock-infected zebrafish at 24 hpi (Fig. 4C). We did not detect a change in* tumor necrosis factor a* mRNA expression in IAV-infected zebrafish compared to PBS-injected zebrafish at 24 hpi (Fig. 4C). Together, our data show that IAV infection activates pro-inflammatory, innate immune cell signaling cascades in zebrafish muscle and culminates in changes to downstream target gene expression.

Next, we infected 2 dpf zebrafish expressing GFP under the control of the neutrophil-specific *myeloperoxidase (mpo or mpx)* promoter^21^ to look at the innate immune response to IAV infection in muscle at the cellular level. In zebrafish at this developmental stage, neutrophils mainly localize to the caudal hematopoietic tissue but can be recruited to sites of injury and/or infection^21^^,^^33^. We also conducted phalloidin staining to allow for visualization of muscle tissue structure. In PBS-injected zebrafish, muscle tissue appeared normal and few GFP-positive neutrophils were observed in muscle (Fig. 4D-D2). Phalloidin staining of IAV-infected zebrafish at 24 hpi revealed sites of damaged muscle fibers, especially in anterior muscle segments (Fig. 4E). Interestingly, neutrophils were found not only to infiltrate muscle tissue in IAV-infected zebrafish (Fig. 4E1), but were observed specifically localized to the unanchored ends of retracted fibers (Fig. 4E2, white arrowheads). The role of neutrophils at the ends of retracted fibers is unknown; however, our data clearly show infiltration of neutrophils in muscle tissue and place neutrophils at the right time and place to play a role in IAV-induced muscle degeneration. Taken together, our data show that inflammation is activated at the molecular and cellular levels in zebrafish muscle in response to IAV infection. Our data also suggest the hypothesis that muscle complications due to IAV infection are may be myositis.

**Molecular and cellular markers of inflammation are present in the muscle tissue of IAV-infected zebrafish:**
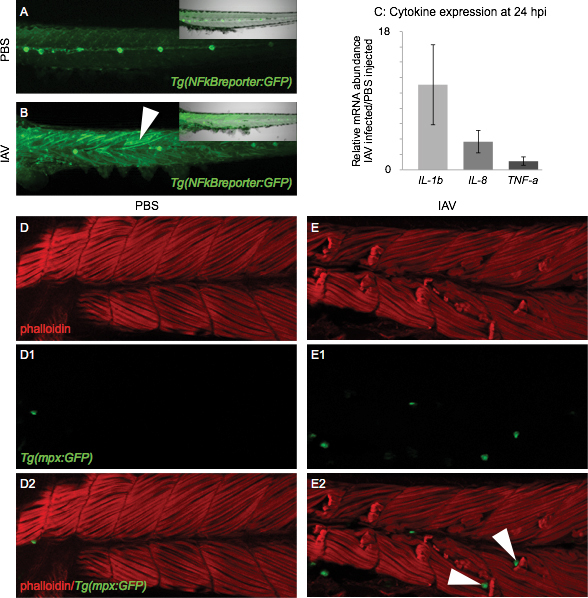
All embryo images are side mounts, dorsal top, anterior left of zebrafish at 24 hpi. (A) PBS-injected Tg(NFkB:GFP) zebrafish embryo which expresses GFP in cells where NFkB transcription factor-dependent gene expression is occurring. NFkB signaling is active in the lateral line system, but not in muscle cells. Inset panel is a merge of fluorescence and brightfield imaging. (B) IAV-injected Tg(NFkB:GFP) zebrafish embryo. NFkB-dependent gene transcription is turned on in muscle cells in response to IAV infection. Inset panel is a merge of fluorescence and brightfield images. (C) Quantification of pro-inflammatory cytokine mRNA expression in IAV-infected zebrafish compared to PBS-injected zebrafish at 24 hpi. Expression of interleukin 1, beta (11.1 +/- 5.2-fold increase; 3 biological replicates; 3 independent experiments) and interleukin 8 (3.7 +/- 1.4-fold increase; 3 biological replicates; 3 independent experiments) increases in response to IAV while tumor necrosis factor a expression remains unchanged (1.1 +/- 0.5-fold increase; 2 biological replicates; 2 independent experiments). (D-E2) Lettered panels show phalloidin staining (red), panels numbered 1 show GFP-positive neutrophils, and panels numbered 2 are merged. (D-D2) PBS-injected Tg(mpx:GFP) zebrafish. Note that not many neutrophils are present in muscle tissue. (E-E2) IAV-infected Tg(mpx:GFP) zebrafish. Note the retracted muscle fibers and the infiltration of muscle tissue by neutrophils. White arrowheads in E2 point to neutrophils localized to the unanchored ends of retracted muscle fibers.

**IAV-infected* sapje/dmd* mutant zebrafish embryos have an increased incidence of muscle damage**

While muscle pain and weakness are unpleasant complications caused by infections, they normally resolve within a week in otherwise healthy individuals. However, severe skeletal or cardiac muscle complications due to infection can occur in immunocompromised patients and can be life-threatening for people with genetic muscle diseases^34^^,^^35^^,^^36^. Skeletal muscle damage is known to be exacerbated in patients with Fukuyama congenital muscular dystrophy upon infection with coxsackie or enteroviruses^34^ or Parvovirus B19^35^. Parvovirus B19 infection in siblings with MDC1A led to severe myocarditis in both patients and was fatal in one case^36^. While individuals with muscular dystrophies are strongly advised to get vaccinated against Influenza viruses due to the potential for respiratory complications, it is unknown, to the best of our knowledge, how IAV infection impacts skeletal muscle tissue in the context of a genetic muscle disease.

To address this question, we infected 2 dpf zebrafish modeling DMD (*sapje/dmd* mutants) with IAV and analyzed muscle tissue structure with phalloidin staining at 24 hpi. Wild-type siblings injected with PBS showed no signs of muscle degeneration in anterior (Fig. 5A) or posterior (Fig. 5B) muscle segments. *Sapje/dmd *mutant embryos injected with PBS displayed foci of muscle degeneration in anterior and posterior muscle segments (Fig. 5C-D, white arrowheads). IAV-infected, wild-type siblings had sites of muscle degeneration mainly in anterior muscle segments (Fig. 5E-F, white arrowhead). *Sapje/dmd* mutant embryos infected with IAV displayed severe muscle degeneration in virtually every muscle segment along the anterior-posterior axis (Fig. 5G-H, white arrowheads). Quantification of the muscle segments with damaged fibers showed that IAV infection and *dmd* mutation have a synergistic effect (i.e. more than an additive effect) in terms of causing muscle degeneration in zebrafish (Fig. 5I). We also tracked survival for 5 days post infection (dpi) (i.e. 7 dpf). PBS-injected *sapje/dmd* siblings or mutants all survived until 5 dpi (Fig. 5J, blue lines). Mortalities were initially observed in IAV-infected, wild-type siblings at 2 dpi, but most mortalities occurred between 4 and 5 dpi (Fig. 5J, green line). There was no difference in total mortality between IAV-infected, wild-type siblings and IAV-infected *sapje/dmd* mutants at 5 dpi; however, mortalities in IAV-infected *sapje/dmd* mutants occurred in greater numbers at earlier time points compared to infected wild-type siblings (Fig. 5J, purple line compared to green line). Our data suggest that underlying *dmd* mutations predispose individuals to muscle damage upon IAV infection and that these individuals are more likely to succumb earlier in the time course of the infection. Altogether, our investigation of skeletal muscle tissue in our zebrafish model of IAV infection shows that muscle is an important *in vivo* target of IAV, that IAV-induced fiber damage is associated with inflammation in muscle tissue, and that muscle complications caused by IAV are greatly exacerbated in a genetic muscle disease model.

**Muscle damage caused by IAV infection is exacerbated in the zebrafish model of DMD:**
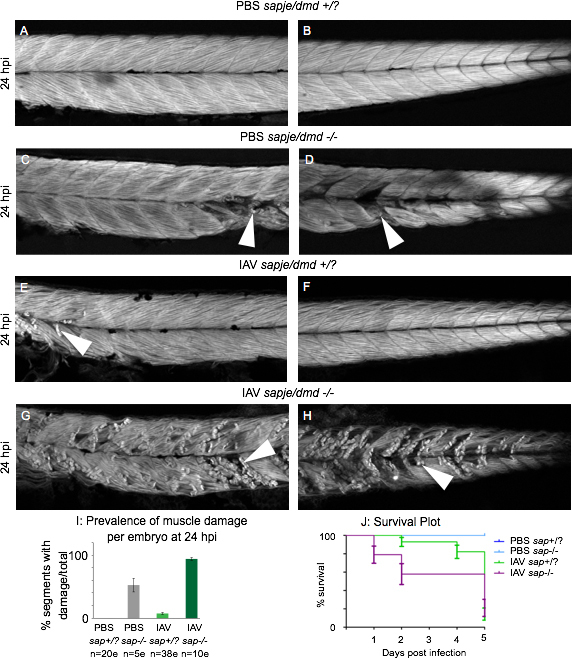
All embryo images are side mounts, dorsal top, anterior left of zebrafish at 24 hpi stained with phalloidin to visualize F-actin. White arrowheads point to foci of muscle damage. (A) Anterior muscle segments of a PBS-injected wild-type sibling embryo. (B) Posterior muscle segments of a PBS-injected wild-type sibling embryo. Note the lack of muscle damage present. (C) Anterior muscle segments of a PBS-injected sapje/dmd mutant embryo. (D) Posterior muscle segments of a PBS-injected sapje/dmd mutant embryo. Note that certain muscle segments in the anterior and the posterior have foci of muscle damage. (E) Anterior muscle segments of an IAV-injected wild-type sibling embryo. (F) Posterior muscle segments of an IAV-injected wild-type sibling embryo. Note the damaged fibers in the anterior muscle segments. (G) Anterior muscle segments of an IAV-injected sapje/dmd mutant embryo. (H) Posterior muscle segments of an IAV-injected sapje/dmd mutant embryo. Note the presence of damaged fibers in every imaged muscle segment of this embryo. (I) Quantification of the average number of muscle segments with damaged fibers per embryo at 24 hpi. Note that the prevalence of fiber damage in IAV-infected sapje/dmd mutants is more than would be predicted from adding the prevalence of damaged fibers of IAV-infected zebrafish and sapje/dmd mutants together. (J) Plot tracking survival for 5 days post injection. All PBS-injected wild-type siblings and sapje/dmd mutants lived for 5 dpi (blue lines). Most mortalities were observed in IAV-infected wild-type siblings between 4 and 5 dpi (green line). IAV-infected sapje/dmd mutants succumbed to the infection earlier than their wild-type siblings with more mortalities occurring on the first and second days post infection. Survival curves from individual experiments representative of three replicates.

## DISCUSSION

Here, we investigated the effects of an infectious disease on skeletal muscle tissue alone and in combination with a genetic muscle disease. We found that human IAV can infect zebrafish muscle fibers and cause fiber damage via loss of sarcolemma integrity and/or loss of ECM adhesion external to the sarcolemma. Additionally, we showed that molecular and cellular markers of inflammation are present in muscle tissue in response to IAV infection. Finally, we showed that an infectious disease in combination with a genetic muscle disease greatly worsens the severity of muscle tissue degeneration. Taken together, our results show that gene-environment interactions are important regulators of muscle tissue structure, function, and health.

We used a model in which transparent embryos/larvae can be infected with a virus that when translated by host cells generates a fluorescent product. This allows for the visualization and tracking of infected cells *in vivo* and in real time. This model could be utilized to extend our findings in ways such as testing the effect of aqueous chemicals on the tropism of IAV for skeletal muscle fibers as well as determining the subcellular localization of GFP puncta in NS1-GFP-infected muscle cells based upon co-localization with fluorescent markers for subcellular organelles. Data from experiments such as these could be used to inform and develop better therapeutic options for preventing and treating skeletal muscle infection by IAV. Inflammation of the heart tissue is another important non-pulmonary complication of IAV infection. Zebrafish infected with IAV display a swollen pericardium, suggesting evidence of viral myocarditis. This model is uniquely suited for the study of viral myocarditis because zebrafish embryos/larvae don’t require heart function due to their small size and aqueous environment where oxygen is freely diffusible for the first 7 dpf^37^. Therefore, zebrafish research could provide insights into the mechanisms underlying viral myocarditis and potential treatments for this condition, which is fatal and thus impossible to study *in vivo* in mammalian models of IAV infection.

We provide evidence that one etiology of muscle fiber death in IAV-infected zebrafish is loss of sarcolemma integrity, similar to dystrophinopathies. This suggests that cytoskeletal disruption may contribute to muscle degeneration upon IAV infection. Influenza virus has been found to associate with and induce changes to the plasma membrane-associated cytoskeleton in infected chick embryo cells^38^. IAV proteins NP and M1 were found to bind to host cell cytoskeletal elements^39^^,^^40^; and, using superresolution or FRET microscopy, the IAV HA protein was recently observed to be localized to actin-rich membrane regions in infected cells^41^^,^^42^. Increased actin polymerization at the plasma membrane of infected cells promotes proper assembly and budding of IAV^43^^,^^44^. The binding of Influenza viral proteins to microfilaments and the reorganization of the cytoskeleton in infected cells, which seems to serve the virus, might be particularly detrimental to skeletal muscle fibers given the critical role of stabilized cytoskeletal-ECM linkages for muscle structure and function. The IAV-induced cytoskeletal disruptions and increased permeability of muscle fibers suggested by our EBD data may be mediated by p38MAPK, Rho/ROCK, and PKC pathways, which have been shown to be involved in changes to the cytoskeleton and permeability in IAV-infected pulmonary microvascular endothelial cells^45^.

A second etiology of fiber death that was found to occur due to IAV infection in zebrafish skeletal muscle tissue is loss of adhesion to the ECM external to the sarcolemma. This could be due to remodeling of the muscle tissue ECM by inflammation. ECM remodeling is achieved via a family of proteinases called matrix metalloproteinases (MMPs). In the lungs of IAV-infected mice, MT1-MMP was found to remodel collagen and blocking MT1-MMP protected infected mouse lungs from tissue damage^46^. Given that laminin-211 is a principle muscle ECM component in zebrafish at the developmental stage of our experiments^28^^,^^47^, it would be interesting to determine the effect of IAV-induced inflammation on the expression and activity of laminin-211-degrading MMPs as well as laminin-211 architecture and binding interactions. Altogether, both IAV- and host-initiated remodeling of the cytoskeleton and the muscle ECM could be mechanisms underlying the muscle fiber damage that we observed to occur in IAV-infected zebrafish.

Finally, our examination of IAV-induced muscle degeneration *in vivo* suggests that muscle complications of IAV are likely caused by a combination of direct infection and inflammation. We detected cellular and molecular markers of inflammation in IAV-infected zebrafish muscle tissue. The inability to consistently detect immune cell infiltration of muscle in IAV-infected humans may be due to varying biopsy locations within and between muscle groups as well as non-standardized timing of biopsies with regards to symptom onset/resolution and infection time course. Our results suggest that anti-Influenza vaccines and other precautionary measures to prevent this infectious disease and the activation of the inflammatory immune response in people (especially those with genetic muscle diseases) is a very important endeavor not just to protect against pulmonary complications of IAV infection, but skeletal muscle damage as well. If IAV is contracted, prompt treatment to reduce muscle tissue inflammation could protect against increased muscle fiber permeability, detachment from the ECM, and fiber death.

## Corresponding Author

Clarissa Henry

Clarissa.Henry@maine.edu

## Data Availability

Our data is up on figshare with the accession number 10.6084/m9.figshare.5499958 <https://doi.org/10.6084/m9.figshare.5499958>. The link is https://figshare.com/articles/Goody_et_al_2017_PLoS_Currents_Muscular_Dystrophy_data/5499958.

## Funding

This work was supported by the National Institutes of Health Grant RO1GM087308 (www.nih.gov/) to C.H.K., the March of Dimes Award number #1-FY14-284 (www.marchofdimes.org) to C.A.H., and the National Institute of Child Health and Human Development Award numbers 5RO3HD077545 and R15HD088217 (www.nichd.nih.gov/) to C.A.H. The funders had no role in study design, data collection and analysis, decision to publish, or preparation of the manuscript.

## Competing Interests statement

The authors have declared that no competing interests exist.
